# Evaluation and Selection of Video Stabilization Techniques for UAV-Based Active Infrared Thermography Application

**DOI:** 10.3390/s21051604

**Published:** 2021-02-25

**Authors:** Shashank Pant, Parham Nooralishahi, Nicolas P. Avdelidis, Clemente Ibarra-Castanedo, Marc Genest, Shakeb Deane, Julio J. Valdes, Argyrios Zolotas, Xavier P. V. Maldague

**Affiliations:** 1 National Research Council Canada, Ottawa, ON K1A 0R6, Canada; Marc.Genest@nrc-cnrc.gc.ca (M.G.); Julio.Valdes@nrc-cnrc.gc.ca (J.J.V.); 2 Computer Vision and Systems Laboratory (CVSL), Department of Electrical and Computer Engineering, Laval University, Quebec City, QC G1V 0A6, Canada; parham.nooralishahi.1@ulaval.ca (P.N.); NP.Avdel@cranfield.ac.uk (N.P.A.); clemente.ibarra-castanedo@gel.ulaval.ca (C.I.-C.); Xavier.Maldague@gel.ulaval.ca (X.P.V.M.); 3 School of Aerospace, Transport and Manufacturing, Cranfield University, Cranfield MK43 0AL, UK; Shakeb.Deane@cranfield.ac.uk (S.D.); A.Zolotas@cranfield.ac.uk (A.Z.)

**Keywords:** active infrared thermography, unmanned aerial vehicle (UAV), composites, aerospace components, video stabilization

## Abstract

Unmanned Aerial Vehicles (UAVs) that can fly around an aircraft carrying several sensors, e.g., thermal and optical cameras, to inspect the parts of interest without removing them can have significant impact in reducing inspection time and cost. One of the main challenges in the UAV based active InfraRed Thermography (IRT) inspection is the UAV’s unexpected motions. Since active thermography is mainly concerned with the analysis of thermal sequences, unexpected motions can disturb the thermal profiling and cause data misinterpretation especially for providing an automated process pipeline of such inspections. Additionally, in the scenarios where post-analysis is intended to be applied by an inspector, the UAV’s unexpected motions can increase the risk of human error, data misinterpretation, and incorrect characterization of possible defects. Therefore, post-processing is required to minimize/eliminate such undesired motions using digital video stabilization techniques. There are number of video stabilization algorithms that are readily available; however, selecting the best suited one is also challenging. Therefore, this paper evaluates video stabilization algorithms to minimize/mitigate undesired UAV motion and proposes a simple method to find the best suited stabilization algorithm as a fundamental first step towards a fully operational UAV-IRT inspection system.

## 1. Introduction

### 1.1. General UAV Applications

The use of Unmanned Aerial Vehicles (UAVs) for the remote inspection of large and/or difficult to access areas has witnessed significant growth in the last few years thanks to their flexibility of movement and their ability to carry multiple sensors. Constant technological evolvement has contributed to making UAVs more affordable, easier and safer to deploy. Moreover, thanks to the recent developments in variety of sensors for UAV applications that are low weight, low power consumption, and improved performance, thereby, allowing multiple sensors to be flown at the same time. UAV related scientific literature is overwhelmingly extensive with a wide variety of applications ranging from precision agriculture [1], traffic analysis [2], 3D mapping/modeling [3], archeological exploration [4], surveillance [5], public safety [6], mining and air pollution monitoring [7], etc. The list is vast and rapidly growing. In some cases, compared to traditional technologies, UAV-based survey systems offer better image spatial resolution (e.g., compared to satellites), and/or are much faster (e.g., compared to ground surveys in remote areas). Moreover, UAV’s flight operation is becoming more automated, and image processing and data fusion tools are continuously evolving [8].

### 1.2. Passive Thermography and UAV Applications

UAVs and Infrared Thermography (IRT) are a perfect match for a contactless survey of thermal phenomena of either large and/or otherwise inaccessible areas, or to ease the inspection of large structures that are difficult to access. The use of UAV-IRT systems has been explored for numerous applications. In most cases, the passive approach has been employed, i.e., the observation of thermal phenomena without the use of external energy stimulation, with the assumption that the features of interests (plants, building materials, photovoltaic cells, people, etc.) will naturally produce thermal gradients that can be isolated from the background. This assumption is valid in many cases, such as building inspection [9] (e.g., detection of thermal bridges, air leakages, moisture, or humidity); precision agriculture [10] (e.g., monitor nutriments levels or lack of water in crop fields); quality assessment of large structures [7] (e.g., inspection of photovoltaic panels farms, wind turbines); or for surveillance applications [11] (e.g., people or animal tracking).

Heat transfer is a complex and transient phenomenon that depends on a combination of factors. This can be used advantageously in some cases, such as for finding the presence of some anomalies (e.g., porosity, fracturing and weathering of rocks, soil slopes, landslide hazard, etc.) [12]. In other cases, the presence of anomalies could be missed if the inspection is not performed at the “correct” time. Building inspection is a good example of this, where the temperature of building materials fluctuates following day/night and seasonal cycles, and also by the effect of weather. Solar Loading Thermography (SLT) [13] exploits the periodic solar irradiation (day and night fluctuations) to retrieve in-depth information about surface and sub-surface anomalies (e.g., cracks, delaminations, thermal bridges, etc.). Moreover, it does so at the expense of large acquisition time, at least 24 h to have a complete “view” of the thermal behavior of different materials.

Another example of passive thermography is to detect water ingression in honeycomb aircraft structures after landing [14], based on the principle that, if water is present inside the honeycomb cells it would take longer to warm-up (thaw) after landing than other materials (aluminum, Nomex, composites) and will appear as cold spots in thermal images.

A different situation is encountered when the feature of interest is at approximately the same temperature as its background. This is the situation found during the inspection of aeronautical components during production or in-service, where typical anomalies can be difficult to detect visually (e.g., cracks, impact damage) and/or can be situated at a certain depth inside the materials (e.g., delaminations, internal damage, liquid ingress). In such cases, the active thermography approach is better suited as explained in the following section.

### 1.3. Active Thermography and UAV Applications

In the case of structures where the features of interest are under the surface and there is no naturally occurring thermal gradient, the passive approach is seldom useful. In those cases, it is far more practical and effective to stimulate the structures to be inspected with a controlled energy source and to use data processing to improve the results [15].

On one hand, the inspection of large structures with the purpose of defect detection and characterization (i.e., determination of the size, depth, or thermo-physical properties) requires careful control of the input energy (duration, waveform type), and data recording (frame rate, time window) to exploit the relationship between the heat transfer rate and the appearance of eventual sub-surface anomalies (shallow defects appear earlier and with superior thermal contrast than deep defects). On the other hand, classical IRT experimental setups allow the inspection of relatively small surfaces at a time (the larger the area the lower the spatial resolution). A map of the complete inspected surface can be reconstructed from several individual inspections (i.e., mosaicking) [16].

Alternatively, large structures can be inspected using a dynamic configuration such as Line Scan Thermography (LST) [17], where the camera and the heat source move in tandem (the camera records thermograms right after heating) with respect to the surface of the component, which is normally static while being inspected. This can be performed for example by mounting a camera and a source on a robot or a 2-axis actuator. LST allows inspecting large and/or complex-shaped components faster than classical static IRT. It is an excellent option for quality control during production. For in-service inspection, the ideal situation would be to inspect an aircraft without the need of removing any component. A LST system would require in this case a huge robotic arm or several smaller robots properly installed and distributed to cover all the areas that need to be inspected. Alternatively, a dynamic system moving “freely” around the aircraft and performing the inspection of all the areas of interest in a fast and effective manner can be conceived. This is where a UAV based active IRT system becomes interesting. Mavromatidis et al. investigated the use of UAVs with active thermography for the inspection and estimation of thermophysical properties of building materials [18]. The authors demonstrated the feasibility of a flash-based system and pointed out the need to improve UAV stability during the acquisition or the development of stabilization post-processing methods.

**Figure 1:**
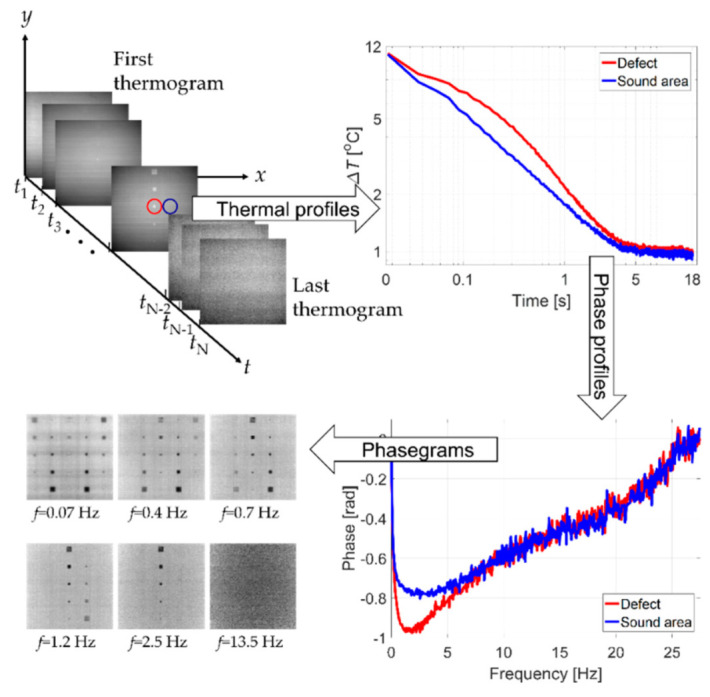
Example of defect detection improvement through signal processing (starting at top left following clockwise motion): raw temperature data sequence; temperature profiles of defective (red) and non-defective (blue) areas; phase profiles of the corresponding areas; and reconstructed phasegrams at selected frequencies, showing the defects.

Although unexpected motions like sudden spikes may have little to no impact on the detection of large and/or shallow damage, detection of small and/or deeper damage often requires further processing. This is illustrated in Figure 1, in which the raw temperature data sequence is processed by pulsed phase thermography or PPT [19] (pixel by pixel through time) to obtain phase profiles that are put together to reconstruct phase images (phasegrams), which significantly improves defect detection. Any undesirable motions can disturb the pixel-wise alignment of consecutive frames, which is already often noisy adding errors in the analysis of temperature evolution used for damage detection. Therefore, video stabilization is required as a first step to minimize/mitigate any undesired motions prior to the application of signal processing technique (e.g., PPT as exemplified in Figure 1), thereby improving the damage detection capabilities of UAV based active thermography inspection technique.

### 1.4. Video Stabilization for UAV Applications

Video stabilization methods are primarily based on mechanical/optical, and digital techniques. In the mechanical/optical stabilization techniques, the camera motion is detected and measured by internal sensors such as accelerometers, gyroscopes, etc. Motion compensation is done by mechanical/optical means, i.e., by using a microcontroller to direct small linear motors to move the image sensor or optically by shifting the lens [20]. Mechanical/optical stabilization is usually built-in as a part of the camera system. Digital video stabilization on the other hand compares the motion between two consecutive frames and shifts the frames to compensate for the undesired motion. The advantages of using digital image stabilization techniques are that there are no moving components and also the ability to apply different algorithms to improve the stabilization.

Digital image stabilization for UAV applications is not new, Shen et al. used block matching technique with polynomial smoothing for stabilization [21]. Wang et al. used corner point detection and matching with a cubic spline for smoothing [22]. In both cases, translations and rotation were used for evaluation. Hong et al. provided a multiresolution video stabilization algorithm based on the Scale Invariant Feature Transform (SIFT) and Haar Wavelet decomposition algorithm. To quantify the improvement, processing time and accuracy were used [23]. Rahmanair et al. used Speeded-up Robust Features (SURF) for motion estimation and Kalman filter to minimize unstable UAV videos used to detect moving objects [24]. Walha et al. used SIFT and Kalman filter with median filter for smoothing and stabilization [25]. Zhou and Ansari [26] compared SIFT and SURF for motion estimation between frames and used Motion Vector Integration (MVI) with adaption damping proposed in [27] for smoothing. Marcenaro et al. used grid and feature-based methods to estimate motion between two consecutive frames [28].

To quantify the stabilized videos Peak Signal to Noise Ratio (PSNR) and Interframe Transformation Fidelity (ITF) were used by Walha, Zhou, Marcenaro, and others, [27,28,29,30,31,32], just to name a few. PSNR and ITF are image quality measurements based on Mean Squared Error (MSE), which is a pixel-by-pixel comparison of two images and does not take into account any changes in luminance and contrast, which are expected to vary due to heating and cooling of the specimen during an active thermography inspection. To evaluate the performance of the video stabilization algorithm Multi-Scale Structural Similarity (MS-SSIM) is used in this work instead of the commonly used PSNR and ITF. MS-SSIM was chosen as it takes into account luminance, contrast, and structural information between two images and compares them at different scales, where in each additional scale the images are passed through a low pass filter and down-sampled by 2 from the previous scale, providing a more advanced image quality measure [31] as compared to PNSR and ITF.

## 2. Experimental Setup

**Figure 2:**
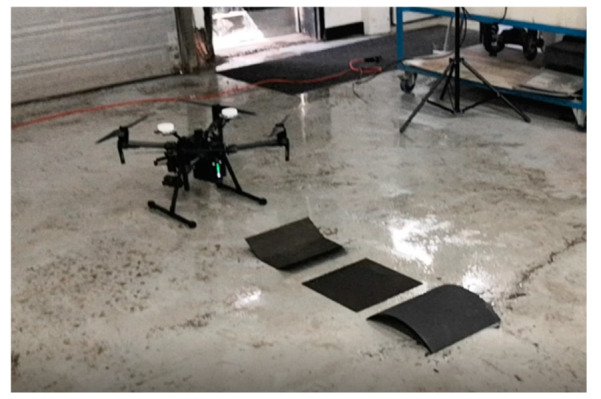
Image of the UAV along with the specimen for the first experimental setup.

Two sets of experiments were performed, where the thermal and optical videos in both experiments were acquired from a DJI Matrice 210 RTK UAV equipped with a Zenmuse X4S (FC6510) optical camera and a Zenmuse XT thermal camera. In the first experiment, the UAV was flown over three carbon fiber specimens that were flat, curved, and trapezoidal-shaped, as shown in Figure 2. The UAV was navigated manually by an experienced pilot in an indoor facility by maintaining a height of approximately 1.5 m above the specimen while acquiring optical and thermal footages at 1920 × 1080 pixels and 720 × 480 pixels respectively for approximately 25 s. The optical and thermal videos were acquired at 24 frames per second (fps) and 30 fps respectively; however, the thermal videos were down-sampled to 24 fps to match the optical videos’ frame rate for future image registration purposes. The first experimental data set was used to develop and validate the video stabilization and selection method.

**Figure 3:**
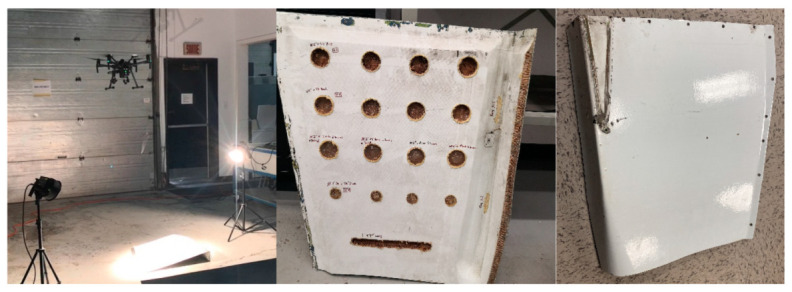
Second experimental setup (**left**), underside defects (**middle**), non-defective side (**right**).

The second experiment was conducted on a Nomex honeycomb core carbon-fiber skin sandwich aircraft part containing manually crafted undersurface defects (holes) at different depths with various shapes and sizes, which are provided in Table 1. The aircraft part was inspected using UAV-based active thermography in an indoor environment using the same UAV and camera setup as the first experiment. The specimen was constantly heated using two halogen flash lamps, as shown in Figure 3. The UAV was navigated manually by an expert pilot, following a predefined flight pattern at three different altitudes of 1.5, 2, and 3 m above the specimen. Only the thermal videos were processed as the purpose of this experimental set was to evaluate a preliminary drone-based active thermography inspection technique, as well as, to further validate the video stabilization algorithm and selection method.

## 3. Methodology

**Figure 4:**
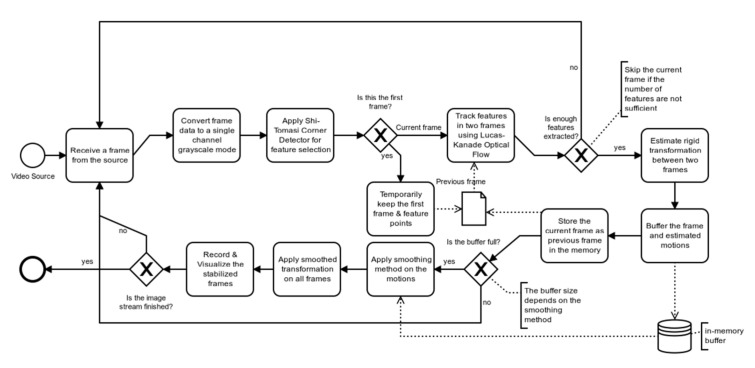
Process flow for video stabilization.

In this paper, a comparative analysis of various smoothing techniques is conducted to develop a method on how to find the most suitable stabilization option for reducing/minimizing the effect of undesired UAV’s motions. For this purpose, the video stabilization process pipeline described in Figure 4 was implemented in Python 3.7.6 with the use of the OpenCV library based on the flow suggested by Thakur [32].

**Figure 5:**
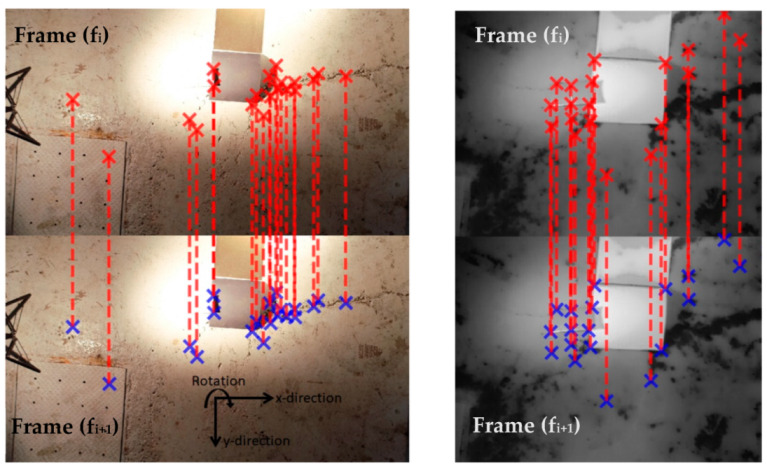
Example of motion estimation using movement of features between two consecutive frames (f_i_) and (f_i+1_) for optical (**left**) and thermal (**right**). Note that the optical and thermal frames shown are not to scale. Optical frames and thermal frames had resolutions of 1920 × 1080 pixels and 720 × 480 pixels respectively.

The process flow shown in Figure 4 can be broken down into seven major steps. First, the desired number of strongest corners or features, shown as (×) in Figure 5 are extracted for frame (f_i_) using Shi-Tomasi method [33]. Second, the extracted features from frame (f_i_) are matched and tracked in the consecutive frame (f_i+1_) using Lucas-Kanade optical flow [34], as shown by dotted lines connecting the features in Figure 5. The number of features is selected such that no frames are skipped. For the first experimental set, 100 features could be reliably tracked for optical videos as compared to 50 features for the thermal videos, which is the result of the lower spatial resolution of the thermal camera capturing fewer details as compared to the optical one, which can also be seen in Figure 5. For both optical and thermal videos, a minimum distance of 20 pixels was set to minimize feature clustering around a single or few strong features. As for the second experiment, 40 features could be reliably tracked with a minimum feature distance set at 20 pixels for all three different heights. Third, an affine transformation matrix is constructed using the features’ movement to find the overall inter-frame motion in the *x*-direction, *y*-direction, and rotation between frames (f_i_) and (f_i+1_). Fourth, all the inter-frame motions are compiled to retrieve the global trajectory of the UAV in the *x*-direction, *y*-direction, and *rotation*. Fifth, different algorithms are used for smoothing the global trajectory. Sixth, individual frames are shifted based on the difference between the original trajectory and the smoothed global trajectory. Seventh, a stabilized video is then constructed from the shifted frames.

**Figure 6:**
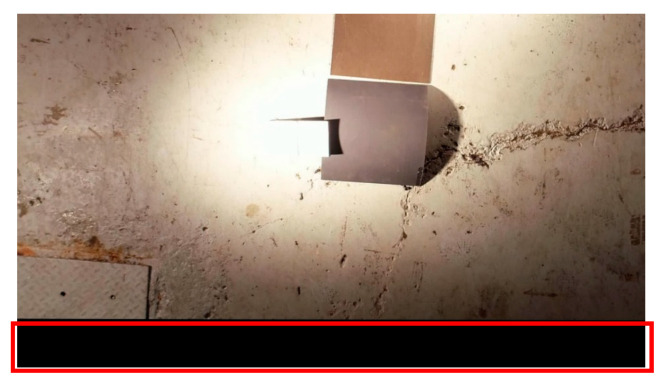
Example of a Blank Border (BB) highlighted within a rectangle. Frame shown is from an optical video with a resolution of 1920 × 1080 pixels.

As a result of shifting a frame for stabilization to fit within the desired video size, undefined regions with black pixels known as Blank Borders (BB), such as the one shown in Figure 6 are generated. For simplicity, optical and thermal stabilized frames from both experimental sets were enlarged to 130% from their initial size to minimize BB in this work.

The BB was calculated for each frame by converting the color image into grayscale first, and then into a binary image by setting the threshold to zero. The binary image was used to find the number of black pixels, which were divided by the overall pixels to find the BB in each frame. The average BB was then calculated to provide information regarding the overall content of BB in the stabilized video.

Several smoothing techniques, such as Simple Moving Average (SMA) [35], Exponential Moving Average (EMA) [35], Gaussian Filter (GF) [36], Linear Regression (LR) [37], Support Vector Regression—Linear Regression (SVR-LR) [38], and a Low-pass Butter worth filter (LBW) [39] were used to stabilize both optical and thermal videos. For SMA and EMA three different window sizes of 1 s (second), 3 s, and 5 s were used. Since both optical and thermal videos were processed at 24 frames per second (fps), 1 s, 3 s, and 5 s refer to window sizes of 24, 72, and 120 frames, respectively. Similarly, for GF, the standard deviation of 24, 72, and 120 frames were used and are referred to as GF-1 s, 3 s, and 5 s, respectively. Default settings were chosen for SVR-LR and LR. As for the LBW, the 5th order with a cut-off frequency of 1 Hz was selected to remove any high-frequency motion. These are the only handful of smoothing techniques with limited parameter settings. Since the focus of this work is to develop a methodology to select an optimal smoothing algorithm for stabilizing UAV based active IRT videos, these algorithms are deemed sufficient to demonstrate the concept.

## 4. Results

**Figure 7:**
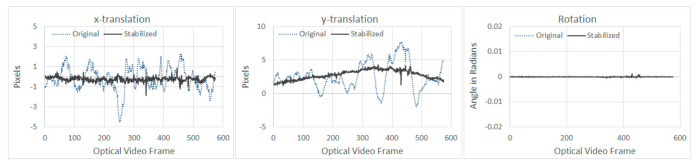
Comparison of extracted motion between original and stabilized frames for optical videos (result shown is that of GF-5s based smoothing).

**Figure 8:**
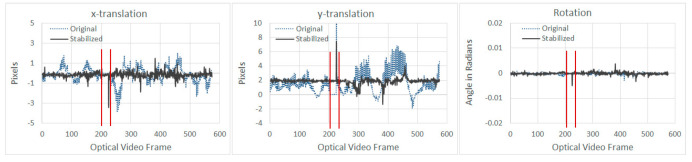
Comparison of extracted motion between original and stabilized frames for thermal videos (result shown is that of LR based smoothing).

For the first experimental run, typical extracted motions between the original and the stabilized frames are shown in Figure 7 and Figure 8 for optical and thermal videos respectively.

From Figure 7, it can be seen that the stabilization algorithm managed to reduce the vibration in both *x* and *y* directions for the optical video. As for the stabilization algorithm results of the thermal video shown in Figure 8, sudden spikes can be seen around frame number 225, which was due to the thermal video being paused while undergoing automatic Flat Field Correction (FFC) for approximately one second [40]. FFC is performed during power up and periodically during operation to compensate for errors, which may have built-up during operation. FFC requires a shutter or similar uniform temperature device to cover the camera field of view [41]. Pausing of the thermal video, while undergoing FFC can be witnessed between frame number 203 to 226, where the *x* and *y*-translations were zero during pausing, followed by a sudden spike (both are highlighted by vertical lines in Figure 8). During this self-calibration period, the thermal camera did not acquire any new frames; however, the UAV continued to fly on its trajectory. The acquisition restarted again upon completion of the FFC process. As can be witnessed from Figure 8, translations and rotation can be prone to outliers caused by sudden shift or lack of enough tracking features between two consecutive frames. To mitigate any errors due to outliers, motions are characterized using Upper Bound (UB) and Lower Bound (LB), which are calculated using Tukey’s fence method, where data outside of LB and UB are considered as outliers [42]. The overall performance of the smoothing techniques for both optical and thermal videos from the first experiment are summarized in Table 2 and Table 3, respectively.

From the summary of the first experimental set provided in Table 2 and Table 3, it can be seen that most of the stabilization algorithms worked well for both optical and thermal videos when average MS-SSIM was used for comparison, i.e., an increase in average MS-SSIM when compared with the original average MS-SSIM. When the actual range of translation was used for comparison, the stabilization algorithm worked equally well for both optical and thermal videos, where there is a reduction in the Range of Motion (RoM) defined as Upper Bound (UB) minus Lower Bound (LB), using some of the algorithms. No significant rotations were present in both optical and thermal videos. It can also be noted that the outlier shown in Figure 8, due to temporary pausing in the thermal video acquisition for auto-calibration did not affect the RoM. As for the BB, the higher the SMA, EMA windows, and GF standard deviations, the higher the BB in the stabilized video. From Table 2 and Table 3, it can also be noticed that some algorithms performed well when average MS-SSIM was used for comparison, while others performed well when RoM and BB were used for comparison. Thus, it is evident that in addition to image quality measure, other parameters should be considered, such as reduction in RoM which provides information regarding how much of the unwanted motion has been reduced, as well as blank border which provides evidence regarding how much the frames are shifted for stabilization, thereby preserving or losing information.

The method proposed in this work takes into account additional features to provide a single metric to evaluate the overall outcome of different stabilization algorithms. The proposed method to identify the best overall algorithm is a weighted Overall Stabilization Metric (OSM). The OSM is based on the Range of MS-SSIM (RoMS_SSIM), defined as the maximum MS-SSIM minus the minimum MS-SSIM ($\max_{\mathrm{MS}\_\mathrm{SSIM}}-\min_{\mathrm{MS}\_\mathrm{SSIM}})$. Additional terms in the OSM are the reduction in the RoM, which is what the stabilization algorithm tries to minimize, and average BB content providing information regarding how much the information in the frames are preserved (0 means no BB, and 1 means that the entire video contains only black pixels). The OSM is expressed as:

(1) $$\mathrm{OSM}\;={\;W}_{MS\_SSIM}\left(\frac{\mathrm{RoMS}\_{\mathrm{SSIM}}_{\mathrm{ori}}-\mathrm{RoMS}\_{\mathrm{SSIM}}_{\mathrm{stab}}}{\mathrm{RoMS}\_{\mathrm{SSIM}}_{\mathrm{ori}}}\right)+\underset{i=x,y,rot}{\sum }W_{i}{\left(\frac{{\mathrm{RoM}}_{\mathrm{ori}}-{\mathrm{RoM}}_{\mathrm{stab}}}{{\mathrm{RoM}}_{\mathrm{ori}}}\right)}_{i}-W_{BB}{\mathrm{BB}}_{\mathrm{average}}$$

where, ori refers to original and stab refers to stabilized. RoM is the Range of Motion and is calculated individually for *x*-translation, *y*-translation and rotation. $W_{MS\_SSIM},\;W_{i=x,y,rot}$, and $W_{BB}$ are weights associated with RoMS_SSIM, individual motion, and BB respectively.

For simplicity, equal weights are assigned for both experimental sets, where out of 100 weight scores, 33.3, 33.3, and 33.4 were assigned to ${\;W}_{MS\_SSIM}$,$\;W_{BB}$, and $W_{x,y,rot}$, respectively. Since there was minimal to no rotation in both experimental sets, $W_{rot}$ was set as zero; whereas, $W_{x}{\;\mathrm{and}\;W}_{y}$ were each assigned 16.7. These weights can be adjusted by the user depending on their preference on what is important. For example, if minimizing BB is important, then the user can assign a higher weight to BB and vice-versa. Results of the OSM for the first experiment are presented in Table 4 for both optical and thermal videos, where OSM values greater than zero would signify overall improvements in the stabilized videos, as compared to the original ones. The greater the OSM value, the better the stabilization algorithm.

From Table 4, it can be seen that for the first experiment most of the smoothing techniques used for the optical video had an overall improvement in the stabilized video (OSM greater than zero); however, some made it worst (OSM less than zero). As for the thermal video, all the smoothing techniques had an overall improvement. The lowest-performing algorithm with the lowest OSM was EMA-5s for both optical and thermal videos (highlighted in Table 4). Upon close inspection from the summary provided in Table 2 for optical video, it can be witnessed that the lowest-performing algorithm significantly increased the RoM instead of reducing them and had significant BB. Similarly, the best performing algorithms with the highest OSM was GF-5s (highlighted in Table 4) for optical video and LR for thermal videos, which had the opposite effect such as: reduction in RoM and range of MS-SSIM, as well as, low to no BB.

**Figure 9:**
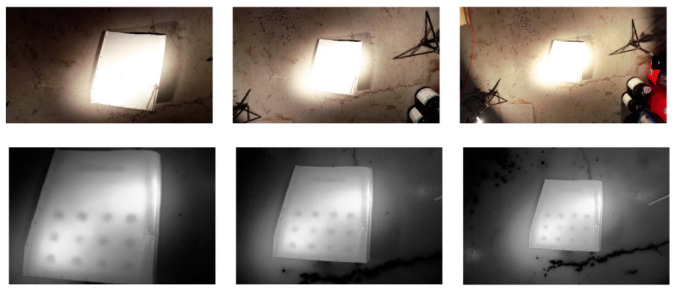
Frames extracted from optical video (**top**) and corresponding thermal video (**bottom**) at increasing UAV heights of 1.5 m, 2 m, and 3 m (from left to right, respectively) during active thermography inspection experiment.

The video stabilization and selection method were further evaluated on the second experimental set, along with a preliminary demonstration of a UAV-based active thermography inspection technique, where the UAV was flown above an aircraft part at three different heights of 1.5, 2, and 3 m, while acquiring optical and thermal videos. Figure 9 shows optical and thermal frames taken at different heights, where the damage (drilled holes) can be seen in the thermal frame but not in the optical frame demonstrating a UAV-based active thermography for detecting damage. As for evaluating the smoothing techniques using the developed OSM approach, the same weight scores that were used in the first experimental set were used here. As mentioned earlier only the thermal video was analyzed from the second experimental set and the outcome is provided in Table 5. For brevity, only OSMs are provided.

**Figure 10:**
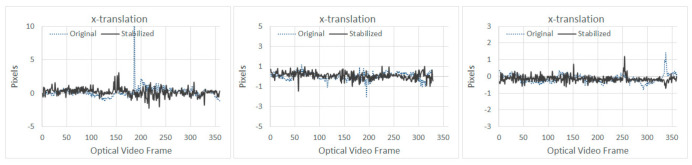
Results of *x*-translation at increasing UAV heights of 1.5, 2, and 3 m (from **left** to **right**) for SVR-LR, LR, and SVR-LR, respectively.

**Figure 11:**
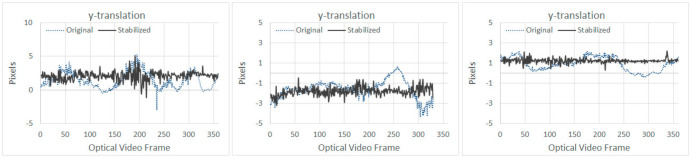
Results of *y*-translation at increasing UAV heights of 1.5, 2, and 3 m (from **left** to **right**) for SVR-LR, LR, and SVR-LR, respectively.

From Table 5 it can be seen that SVR-LR provided the best overall results, i.e., the highest OSM value when the UAV was flown at heights above the specimen at 1.5 and 3 m; whereas, LR provided the best results when the UAV was flown at 2 m (highlighted in Table 5). This also highlights that since UAV motions are unpredictable, a single smoothing technique may not always provide the best results even for similar applications. Results of the stabilization in *x* and *y* translations are provided in Figure 10 and Figure 11, respectively for the best performing algorithms at different heights.

The presence of an outlier in the *x*-translation can be seen in (Figure 10 left), since Tuckey’s fencing technique [42] is adopted for OSM, outliers like these have no effect in the OSM calculation. No significant improvements can be noted in the *x*-translation (Figure 10); however, for *y*-translation (Figure 11), the stabilization algorithm managed to reduce the overall range of motion, which was also observed during the first experiment, as shown in Figure 7 and Figure 8.

## 5. Discussion

The video stabilization and selection method presented in this paper was developed for UAV based active thermography inspection, where it was deemed essential to improve the damage detection capabilities. The video stabilization method was based on the one suggested by Thakur [32]. In terms of quantifying the improvement of the stabilized videos, several new methods are proposed. First, instead of using PSNR and ITF, the range of MS-SSIM is used because MS-SSIM considers luminance, contrast, and structural information between two images and compares them at different scales, providing a more advanced image quality measure. Additionally, active thermography relies on processing temperature evolution over time of acquired image sequence; therefore, MS-SSIM range provides a better indication of how close or far-apart all the images are to one another—the smaller the range of MS-SSIM, the more similar the images in the video are to one another; and hence, the better the stabilization algorithm. Second, the inclusion of RoM, which is the difference between Tuckey’s fence UB and LB capturing the reduction in the overall motion while removing any outliers, providing a robust method to quantify stabilization. Third, the inclusion of BB, which is created due to excessive shifting of the frames for stabilization, offering a quantitative indication of how much of the information is retained or lost due to shifting.

It was also found that the majority of the research in the literature was conducted on a single or handful of stabilization algorithms, where the improvements were quantified using individual comparison metric such as translations, rotations, processing time, accuracy, PSNR, ITF, etc. In this work, the range of MS-SSIM, reduction in undesired motion, and BB were all included in a single quality metric to provide a complete evaluation of various stabilization algorithms. The stabilization and selection methods were applied to optical and thermal videos from two different experimental sets; the outcome of which is provided in the Results section. The highest scoring OSM was selected as the best performing algorithm. Enhancements from the higher scoring OSM algorithms can be witnessed as a significant reduction in the RoM, lower MS_SSIM ranges, and low BBs, all of which signify overall improvements in the stabilized videos.

The OSM calculation contains BB, which can be further reduced by enlarging the stabilized frame size; however, this can result in information loss and, therefore, care must be taken. There are ways to fill these BBs using several techniques such as mosaicking [43], finding a match in the neighboring frames [44], interpolating matching sharper pixels from the neighboring frames for stitching [45], etc. These techniques to fill the BB, are applicable to optical videos but may not be applicable to thermal videos used for pulse/step-heating based active thermography inspection because they rely on the pixel temperature evolution over time, which is expected to vary between neighboring frames. The OSM presented in this paper neatly captures important aspects of video stabilization and provides a simple means to evaluate different algorithms to identify the best one. The user can also add processing time to the OSM, if it is deemed critical.

It can also be noted that in the second experiment the specimen was heated constantly using halogen lamps for active thermography inspection, therefore damages can be seen in the thermal frames although the depth and sizes of these damages are difficult to determine. To detect and size the damage at different depths, infrared thermography post-processing techniques can be employed [46], where the pixel-wise thermal evolution is processed using several techniques such as pulsed phase thermography, shown in Figure 1 [19], thermographic signal reconstruction (TSR) [47], TSR combined with 1st and 2nd derivative approaches [48], principal component analysis [49], etc. Methods to stabilize and select an optimal video stabilization such as the one presented in this paper are required for such applications to reduce/eliminate any pixel shifts attributing to the post-processing errors. Continuation of this work includes developing a UAV-based pulse/step-heating inspection technique and applying the methods developed here to stabilize and select the optimal smoothing techniques.

## 6. Conclusions

An Unmanned Aerial Vehicle (UAV) based inspection system that can move “freely” around an aircraft to perform the inspection of all the areas of interest in a fast and effective manner can have a significant impact in reducing inspection time and cost. However, UAV inspection is challenging because the UAV carrying the optical and thermal cameras is subject to vibration and undesired motion. To reduce such undesired motion, a digital video stabilization technique along with a proper methodology to select the best smoothing techniques are presented in this paper. The stabilization method is based on finding the motion between two consecutive frames using a features-based approach. To evaluate the performance of the video stabilization algorithms Multi-Scale Structural Similarity (MS-SSIM), reduction in undesired motion, and Blank Border (BB) were used. Some algorithms performed better when MS-SSIM was used for comparison, while others performed better when the range of motion and BB were used for comparison. Instead of using three different comparison metrics, a simple weighted Overall Stabilization Metric (OSM) based on the reduction in the range of MS-SSIM and motion, as well as average BB content was proposed for an overall evaluation of the stabilization algorithms. The stabilization and selection methods were evaluated on two different experimental sets. The first experimental set was used to develop and test the methodologies; whereas, the second experiment was conducted to demonstrate a UAV-based active thermography technique, as well as, to evaluate the methods developed to stabilize and select the best smoothing techniques. The OSM showed that different smoothing techniques had different stabilization results, some improved them, and some made them worst. The highest OSM was used as a criterion to find the best-suited algorithm. The highest scoring smoothing techniques all had low range of MS-SSIM and motions, as well as, low BB content, all of which are characteristics of better overall stabilization. Therefore, the method presented in this paper provides a simple means to stabilize videos and to evaluate different stabilization algorithms to select the one that is best suited for the application, which is a fundamental first step towards developing a fully operational UAV-based active thermography inspection system.

## Figures and Tables

**Table 1:** Aircraft part with underside defects placed in five rows and are partly filled with silicone to simulate different depth.

Rows	Defect Type	Quantity	Size	Depth
1	Circular	4	5 cm (diameter)	2.2 cm
2	Circular	4	5 cm (diameter)	1 cm (filled)
3	Circular	4	5 cm (diameter)	(filled)
4	Circular	4	5 cm (diameter)	(filled)
5	Rectangular	1	2 × 24 cm	2 cm

**Table 2:** Summary of optical video stabilization using different trajectory smoothing techniques for first experimental set.

Smoothing Algorithm	x-Translation (Pixels)		y-Translation (Pixels)		Rotation (Radians)		MS-SSIM			Average BB Percent
	Lower Bound	Upper Bound	Lower Bound	Upper Bound	Lower Bound	Upper Bound	Min	Max	Average	
**Original**	−4.54	2.28	−1.98	7.74	0.00	0.00	0.72	1.00	0.86	0.00
**EMA-1s**	−3.18	1.50	0.25	10.97	0.00	0.00	0.75	0.99	0.88	0.08
**EMA-3s**	−4.32	1.42	−0.97	8.55	0.00	0.01	0.80	0.99	0.90	4.85
**EMA-5s**	−24.55	1.36	0.00	42.68	0.00	0.00	0.84	0.99	0.92	12.67
**GF-1s**	−3.08	1.81	1.31	7.86	0.00	0.00	0.74	0.96	0.88	0.00
**GF-3s**	−1.06	1.14	1.33	5.00	0.00	0.00	0.81	0.95	0.88	0.00
**GF-5s**	−1.87	0.85	1.22	4.29	0.00	0.00	0.83	0.96	0.90	0.20
**LBW**	−5.94	2.65	−2.42	10.43	0.00	0.00	0.71	1.00	0.87	0.00
**LR**	−1.23	5.00	0.68	4.20	0.00	0.00	0.78	0.91	0.86	0.12
**SMA-1s**	−4.71	2.05	−1.20	9.94	0.00	0.00	0.73	0.99	0.87	0.00
**SMA-3s**	−3.13	6.10	0.03	9.94	0.00	0.00	0.70	0.99	0.88	1.05
**SMA-5s**	−2.28	1.40	−0.04	7.64	0.00	0.00	0.75	0.99	0.88	3.56
**SVR-LR**	−0.92	7.71	0.74	4.19	0.00	0.00	0.79	0.91	0.86	0.34

**Table 3:** Summary of thermal video stabilization using different trajectory smoothing techniques for first experimental set.

Smoothing Algorithm	x-Translation (Pixels)		y-Translation (Pixels)		Rotation (Radians)		MS-SSIM			Average BB Percent
	Lower Bound	Upper Bound	Lower Bound	Upper Bound	Lower Bound	Upper Bound	Min	Max	Average	
**Original**	−1.74	1.55	−1.90	4.59	0.00	0.00	0.86	1.00	0.97	0.00
**EMA-1s**	−1.37	1.11	−0.46	3.71	0.00	0.00	0.92	1.00	0.98	0.40
**EMA-3s**	−1.14	0.88	−0.48	3.99	0.00	0.00	0.89	1.00	0.98	8.64
**EMA-5s**	−1.09	0.83	−0.74	3.99	0.00	0.00	0.85	1.00	0.99	19.33
**GF-1s**	−1.29	0.99	−0.07	3.45	0.00	0.00	0.92	1.00	0.98	0.00
**GF-3s**	−0.76	0.45	0.16	3.34	0.00	0.00	0.93	0.99	0.98	0.10
**GF-5s**	−0.70	0.42	0.33	2.93	0.00	0.00	0.93	0.99	0.99	0.55
**LBW**	−2.34	2.14	−2.00	5.49	0.00	0.00	0.92	1.00	0.98	0.00
**LR**	−0.70	0.39	1.37	2.45	0.00	0.00	0.92	0.99	0.98	0.57
**SMA-1s**	−1.90	1.71	−1.36	4.74	0.00	0.00	0.92	1.00	0.98	0.00
**SMA-3s**	−1.38	1.10	−0.36	3.57	0.00	0.00	0.92	1.00	0.98	2.05
**SMA-5s**	−0.98	0.79	−0.77	4.65	0.00	0.00	0.93	1.00	0.98	6.64
**SVR-LR**	−0.73	0.35	1.26	2.43	0.00	0.00	0.88	0.99	0.98	1.05

**Table 4:** Summary of OSM for different smoothing algorithm for first experimental set.

Smoothing Algorithm	Optical Video OSM	Thermal Video OSM
**Original**	0.00	0.00
**EMA-1s**	7.92	25.34
**EMA-3s**	11.80	16.15
**EMA-5s**	**−92.48**	**2.64**
**GF-1s**	17.28	29.12
**GF-3s**	37.85	37.09
**GF-5s**	**39.84**	39.63
**LBW**	−10.64	5.23
**LR**	30.52	**42.26**
**SMA-1s**	−0.57	14.22
**SMA-3s**	−8.26	24.76
**SMA-5s**	14.47	24.33
**SVR-LR**	25.15	31.60

**Table 5:** Summary of OSM for different smoothing techniques applied to the thermal video from the second experimental set, where the UAV was flown at three different heights.

Smoothing Algorithm	UAV Flown at 1.5 m Height OSM	UAV Flown at 2 m Height OSM	UAV Flown at 3 m Height OSM
**Original**	0.00	0.00	0.00
**EMA-1s**	−18.90	14.53	17.46
**EMA-3s**	0.38	13.28	27.57
**EMA-5s**	−2.15	0.86	29.51
**GF-1s**	14.15	28.98	25.75
**GF-3s**	18.92	25.37	38.20
**GF-5s**	8.33	23.60	29.75
**LBW**	−19.82	−4.14	−4.66
**LR**	20.24	**33.97**	43.82
**SMA-1s**	−10.61	3.03	2.07
**SMA-3s**	−29.68	18.08	10.40
**SMA-5s**	−6.69	10.32	20.54
**SVR-LR**	**22.93**	32.79	**44.75**

## Data Availability

The data presented in this study are available on request from the corresponding author.
